# Interfacial Charge Transport Engineering in MoO_3_ via Integration of PEDOT:PSS for Efficient and Durable Oxygen Evolution Reaction

**DOI:** 10.3390/molecules31213803

**Published:** 2026-10-22

**Authors:** Mrunal Bhosale, Sahil Sanjay Magdum

**Affiliations:** School of Chemical Engineering, Yeungnam University, Gyeongsan 38541, Gyeongsanbuk-do, Republic of Korea

**Keywords:** oxygen evolution reaction, conductive polymer interface, MoO_3_-PEDOT:PSS hybrid electrocatalyst, electrode–electrolyte interface, interfacial charge transport

## Abstract

Electrochemical water oxidation is a grave half-reaction in overall water splitting. However, its practical efficiency is largely restricted by sluggish oxygen evolution reaction (OER) kinetics and the poor electrical conductivity of many transition-metal oxide catalysts. Herein, we report a rationally engineered molybdenum oxide-poly(3,4-ethylenedioxythiophene) (MoO_3_-PEDOT:PSS) hybrid electrocatalyst designed to enhance interfacial charge transport and accelerate alkaline OER kinetics. The MoO_3_ framework was first grown on carbon cloth through a hydrothermal route, accompanied by PEDOT:PSS surface modification using a simple immersion strategy. The conformal deposition of PEDOT:PSS onto molybdenum oxide nanostructures establishes a highly conductive and electronically coupled interface that significantly accelerates charge transport and optimizes adsorption energetics of reaction intermediates. Benefiting from the optimized integration of conductive PEDOT:PSS with the MoO_3_ framework, the MoO_3_-PEDOT:PSS-2 electrode delivers enhanced OER activity with a low overpotential of 302.4 mV at 10 mA cm^−2^, a Tafel slope of 65.9 mV dec^−1^, and stable performance after 5000 CV cycles and 30 h chronopotentiometry. In particular, the PEDOT:PSS layer not only facilitates efficient electron transport but also stabilizes the reconstructed active phases during prolonged operation, thereby suppressing performance degradation. This work highlights the critical role of polymer-assisted interfacial electronic modulation in transition metal oxides and provides a versatile strategy for designing next-generation, binder-free electrocatalysts for effectual and durable water oxidation.

## 1. Introduction

Due to the increasing energy demand worldwide as well as the grave environmental impacts of fossil fuel combustion, the progress of clean, efficient, and sustainable energy technologies has developed an important research topic for researchers. Hence, extensive endeavors have been put forward to develop systems based on renewable energy technologies capable of large-scale energy conversion and storage [1,2,3]. Within the area of electrochemical energy technologies, the oxygen evolution reaction (OER) and the hydrogen evolution reaction (HER) have been recognized as two fundamental and potential approaches toward clean energy conversion and storage especially in water splitting [4,5,6]. Anodic OER and cathodic HER are the key processes to produce hydrogen that has been identified as one of the clean and renewable energy carriers [7,8]. The oxygen evolution reaction is a complex four-electron oxidation process that involves sluggish reaction kinetics and a high thermodynamic/kinetic energy barrier, which significantly limits the efficiency of overall water splitting [9]. Recently, transition-metal-based composite oxides are becoming highly attractive as cost-effective and earth-abundant candidates with tunable electronic properties and wide applications in water electrolysis [10,11], Li-ion batteries [12], solar cells and the like, due to their unique properties. Nevertheless, the total efficiency of water splitting is primarily limited by the poor kinetics of the OER. The OER requires a complex four-electron transfer process to produce one oxygen molecule, while HER involves only a two-electron transfer process, so the former is the rate-determining step of water electrolysis [13]. Therefore, the OER usually requires a significantly higher overpotential than the HER, restricting the commercial application of hydrogen production via electrochemical water splitting [14,15].

Noble-metal-based catalysts such as RuO_2_ and IrO_2_ display excellent OER activity; however, their high cost, scarceness, and limited long-term stability restrict their large-scale practical application [16,17,18,19]. As a result, transition-metal oxides, hydroxides [20], sulfides [21], phosphides [22], and molybdate-based materials have attracted significant attention as alternative OER catalysts [23]. Among them, molybdenum oxide (MoO_3_) is a promising candidate owing to its multiple oxidation states, layered crystal structure, good chemical stability, and rich redox chemistry. The presence of Mo^6+^/Mo^5+^ redox couples can facilitate surface charge transfer and promote electrochemical reactions [24,25,26]. Co_3_(PO_4_)_2_-MoO_3-x_ for alkaline water/seawater splitting [27], ZIF67@MoO_3_ NSs@NF composite for effective oxygen evolution reaction and overall water-splitting [28], IrO2/MoO_3_ [29], MoO_3_ patches into NiNS [30]. However, the practical OER performance of pristine MoO_3_ is still limited by its poor electrical conductivity, restricted active-site exposure, and sluggish interfacial charge transport.

To overcome these limitations, coupling MoO_3_ with conductive polymers is an effective strategy to improve its electrocatalytic activity. Conductive polymers can enhance electron transport, regulate surface morphology, and provide flexible interfacial pathways for charge transfer [31,32,33]. Among various conductive polymers, poly(3,4-ethylenedioxythiophene), commonly known as PEDOT:PSS, is particularly attractive because of its high electrical conductivity, excellent environmental stability, good adhesion, and favorable electrochemical properties [34,35]. For example, Zhang et al. reported that an rGO/PEDOT:PSS composite can serve as an efficient catalyst for the oxygen reduction reaction, where the incorporation of PEDOT:PSS enhanced the electrical conductivity of the composite and improved its resistance toward CO poisoning [36]. Bingyang Ma et al. developed an MXene@PEDOT composite as a highly efficient photoelectrocatalyst for water-splitting applications [37]. Baruah B et al. developed NiO composited with PEDOT:PSS and rGO bifunctional electrocatalyst for methanol oxidation and OER [38].

**Figure 1:**
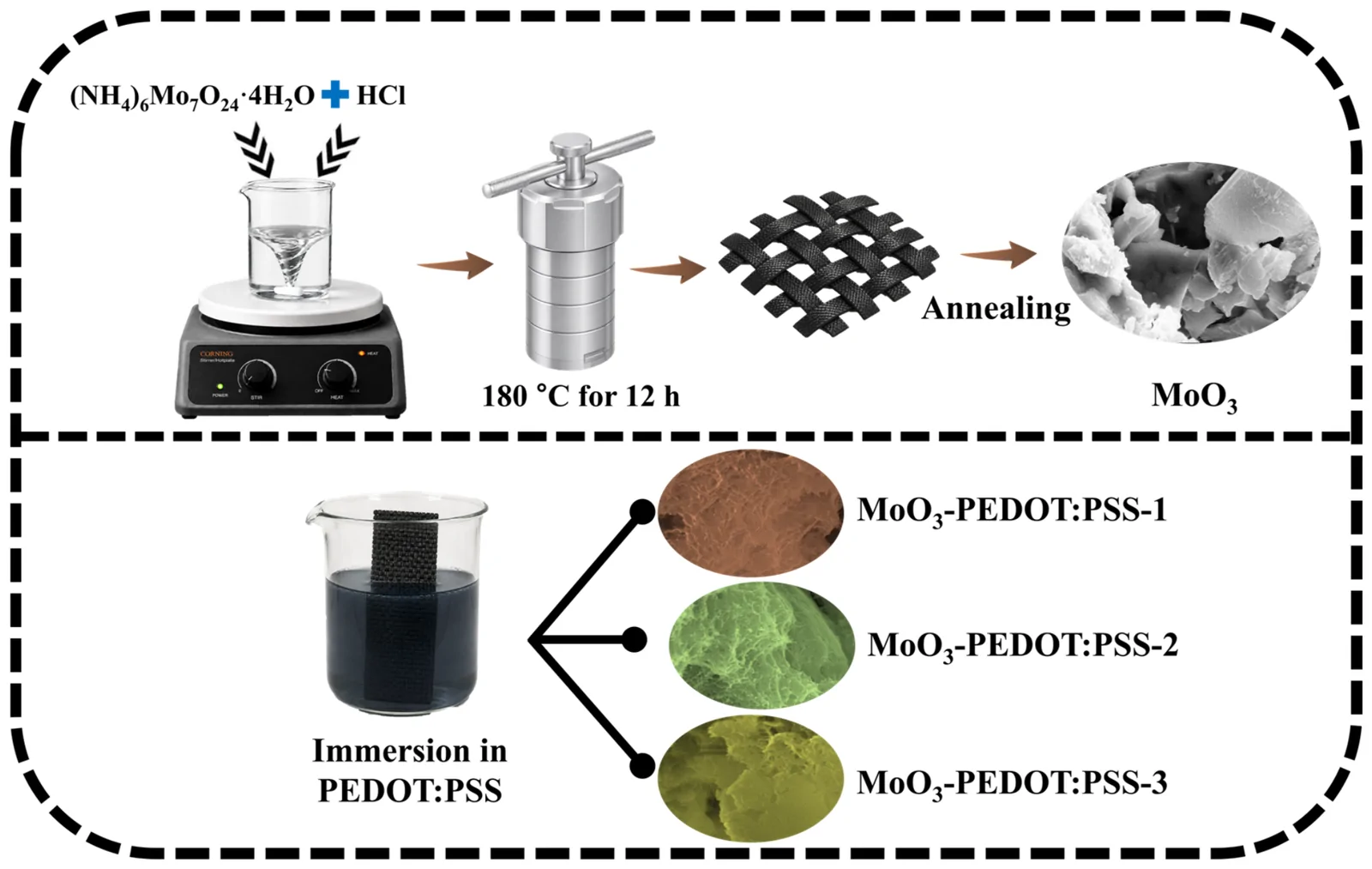
Schematic illustration of MoO_3_-PEDOT:PSS synthesis.

The incorporation of PEDOT:PSS with MoO_3_ can create a synergistic hybrid structure in which MoO_3_ provides abundant redox-active sites, while PEDOT:PSS acts as a conductive network that accelerates electron transfer and improves electrode/electrolyte interaction. In this work, we demonstrate the synthesis of MoO_3_-PEDOT:PSS hybrid electrocatalysts (Figure 1) for the enhanced OER in an alkaline medium and investigate their electrochemical performance. The optimized MoO_3_-PEDOT:PSS electrode showed a high OER performance than pure MoO_3_ in terms of low overpotential of 302.4 mV, rapid Tafel slope of 65.9 mV.dec^−1^, reduced charge-transfer resistance (R_ct_) of 4.6 Ω, and an advanced electrochemically active surface area of 103.87 cm^2^. These performances are attributed to synergistic interactions between MoO_3_ and PEDOT:PSS, that can contribute to increasing the conductivity of PEDOT:PSS, enhancing the interfacial coupling and charge transport at the interface of the electrode and electrolyte.

## 2. Result and Discussion

**Figure 2:**
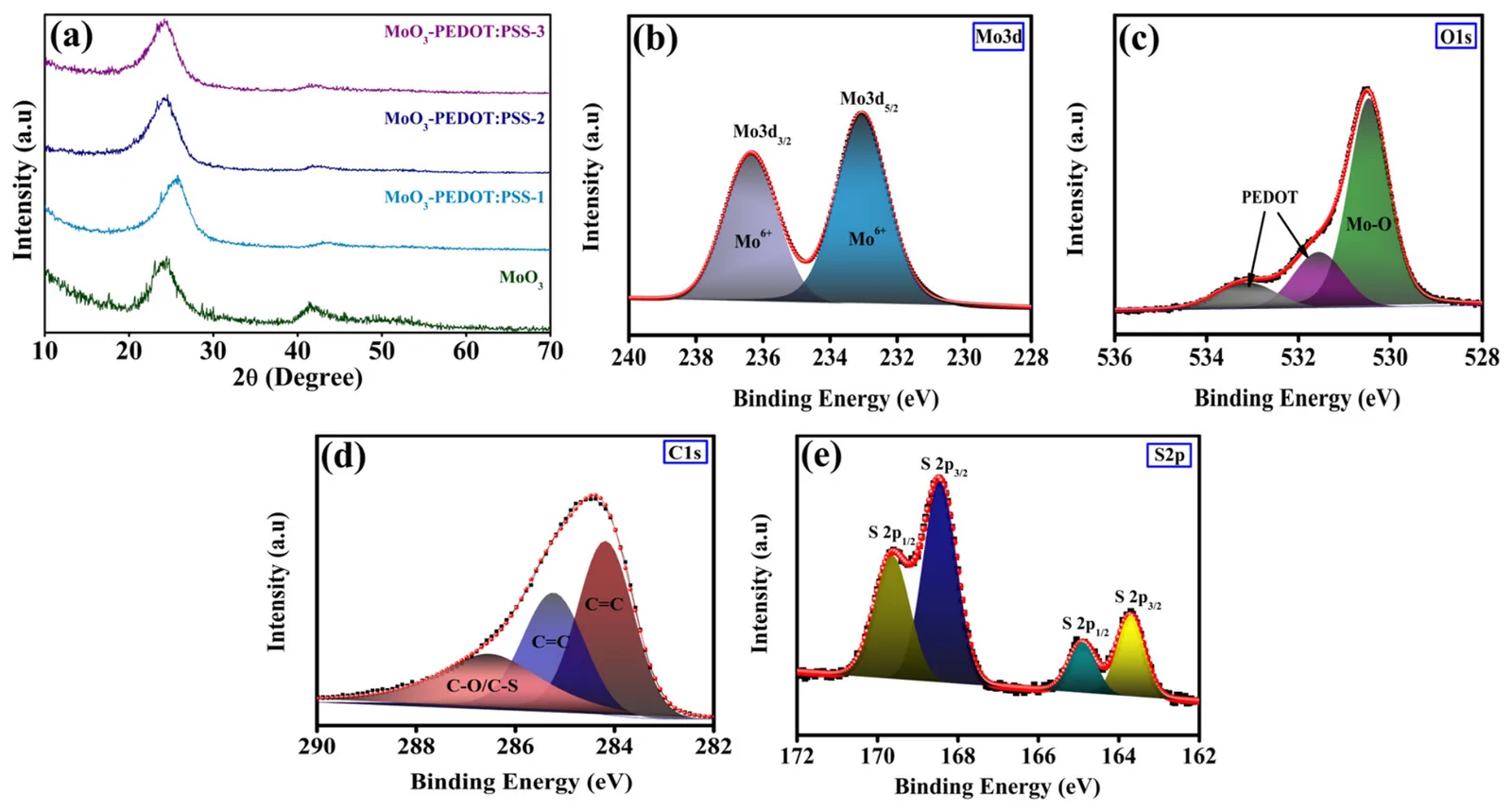
(**a**) XRD spectra of all the materials. High-resolution XPS spectra, (**b**) Mo 3d, (**c**) O 1s, (**d**) C 1s, and (**e**) S 2p spectra of MoO_3_-PEDOT:PSS-2 composite.

The crystalline phase and structural evolution of MoO_3_ and MoO_3_-PEDOT:PSS composite electrodes were examined by X-ray diffraction (XRD), as exposed in Figure 2a. The XRD profiles recorded in the 2θ range of 10–70° display the characteristic diffraction features of MoO_3_ along with the contribution from the carbon cloth substrate. For pristine MoO_3_, the diffraction peaks situated at 2θ values of 17.6°, 20.7°, 23.0°, 28.7°, 29.9°, 33.8°, 34.9°, and 53.5° can be indexed to the (011), (110), (020), (−211), (111), (−222), (022), and (310) crystallographic planes of MoO_3_, respectively, confirming the effective formation of the MoO_3_ phase. These diffraction reflections are consistent with the standard JCPDS card No. 03-065-7675. The occurrence of distinct diffraction peaks directs the crystalline nature of the synthesized MoO_3_ structure. In addition to the MoO_3_-related reflections, broad diffraction bands centered around 24.9° and 42.4° are observed, which correspond to the (002) and (100) planes of carbon cloth, respectively. These peaks are in good agreement with JCPDS card No. 75-1621 [39]. After the introduction of PEDOT:PSS, the overall diffraction profile of MoO_3_ is retained, suggesting that the PEDOT:PSS deposition does not destroy the primary crystalline framework of MoO_3_. However, no additional sharp diffraction peaks related to PEDOT:PSS are detected in the MoO_3_-PEDOT:PSS composites, indicating that PEDOT:PSS is mainly present in an amorphous or poorly crystalline state on the MoO_3_ surface [40,41]. Notably, with increasing PEDOT:PSS incorporation, the intensity of the MoO_3_ diffraction peaks gradually declines. This attenuation can be attributed to the progressive surface coverage of crystalline MoO_3_ by the amorphous PEDOT:PSS layer, which weakens the diffraction signal originating from MoO_3_. Meanwhile, the broad peak near 24–25° becomes more pronounced after PEDOT:PSS deposition, which may arise from the overlapping contribution of the carbon cloth substrate and the amorphous/conjugated polymeric structure of PEDOT:PSS. This observation further supports the successful formation of the MoO_3_-PEDOT:PSS composite. The XRD results confirm the successful integration of amorphous PEDOT:PSS with crystalline MoO_3_, forming a MoO_3_-PEDOT:PSS composite structure while preserving the fundamental MoO_3_ phase.

X-ray photoelectron spectroscopy (XPS) was carried out to examine the surface chemical composition, and elemental valence states of the optimized MoO_3_-PEDOT:PSS-2 composite. The high-resolution Mo 3d spectrum, shown in Figure 2b, displays two noticeable peaks located at binding energies of 233.1 and 236.3 eV. These peaks are allocated to Mo^6+^ 3d_5/2_ and Mo^6+^ 3d_3/2_, respectively, confirming the predominant presence of Mo in the +6-oxidation state within the MoO_3_ framework [42,43]. The clear spin–orbit splitting of the Mo 3d doublet further verifies the successful formation of MoO_3_ in the composite structure. The O 1s spectrum of MoO_3_-PEDOT:PSS-2, presented in Figure 2c, was deconvoluted into three oxygen-related components. The peak positioned at 530.4 eV corresponds to lattice oxygen related with Mo-O bonding in MoO_3_. The additional peaks situated at 531.5 and 533.2 eV can be ascribed to oxygen-containing functional groups associated with PEDOT:PSS [44,45,46]. Figure 2d presents the high-resolution C 1s spectrum of the MoO_3_-PEDOT:PSS-2 composite. The deconvoluted peak at 284.1 eV is credited to the C=C bonding environment associated with the α-position carbon atoms of the thiophene ring, while the peak at 285.2 eV resembles to the C=C bonding of β-position carbon atoms in the PEDOT:PSS backbone. The additional peak located at 286.5 eV is allocated to C-O/C-S bonding, originating from the ethylenedioxy groups and thiophene units of PEDOT:PSS [47,48,49]. Furthermore, the high-resolution S 2p spectrum (Figure 2e) confirms the sulfur-containing thiophene structure of PEDOT and sulfonate structure in PSS chain. Two characteristic peaks located at 163.7 and 165.3 eV are assigned to S 2p_3/2_ and S 2p_1/2_, respectively, corresponding to sulfur atoms in the PEDOT:PSS backbone [37,50]. The additional peaks located at 168.4 and 169.6 eV can be assigned to the S 2p_3/2_ and S 2p_1/2_ spin–orbit components of sulfonate sulfur species in the PSS chain, respectively [49,51]. The appearance of these sulfur signals, along with the C 1s and O 1s contributions from PEDOT:PSS, confirms the successful deposition of PEDOT:PSS onto MoO_3_.

**Figure 3:**
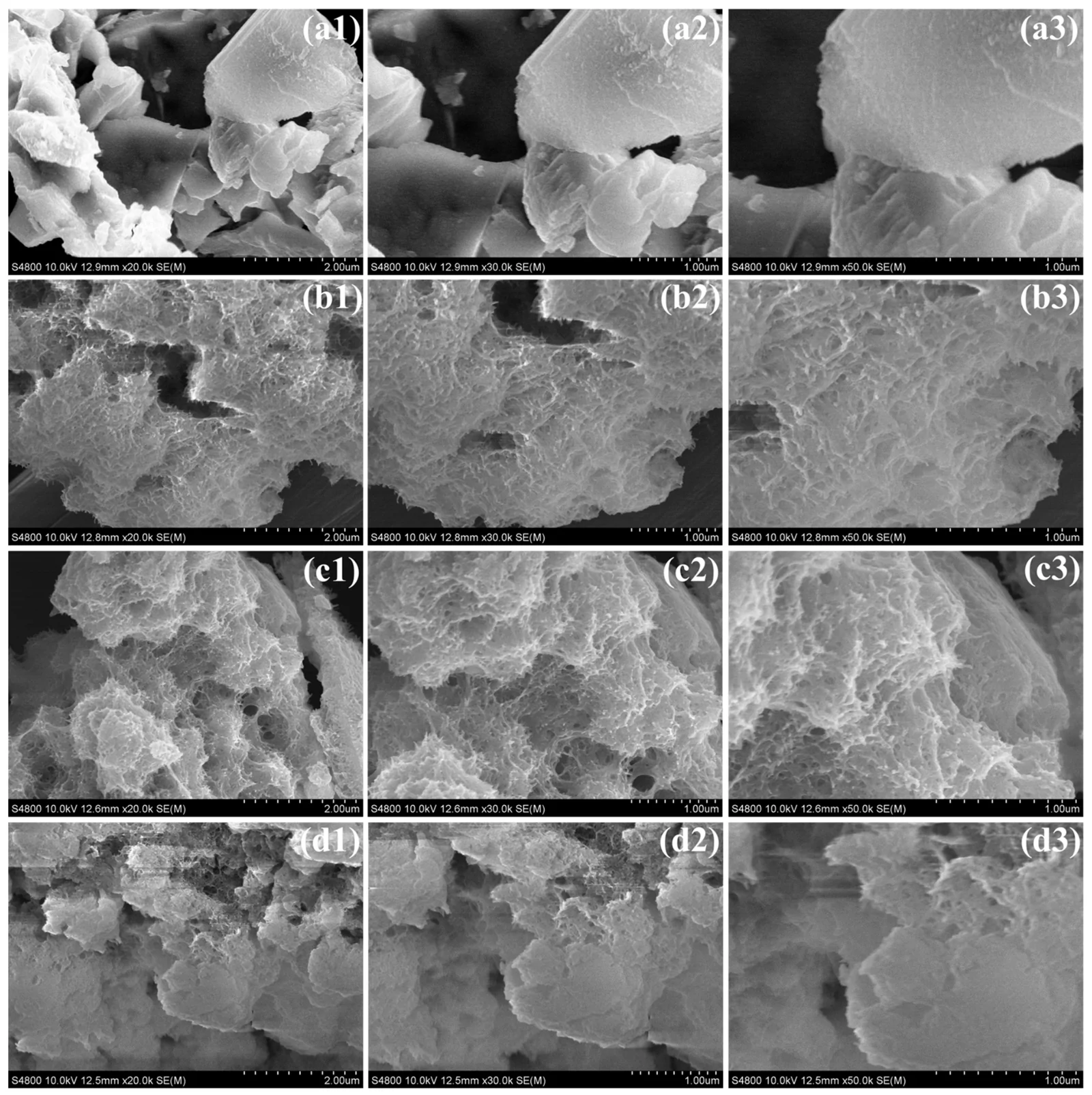
SEM micrograph images of (**a1**–**a3**) MoO_3_, (**b1**–**b3**) MoO_3_-PEDOT:PSS-1, (**c1**–**c3**) MoO_3_-PEDOT:PSS-2, and (**d1**–**d3**) MoO_3_-PEDOT:PSS-3.

The surface features and morphological development of the MoO_3_ and MoO_3_-PEDOT:PSS composite electrodes were examined using FESEM at various magnifications, as presented in Figure 3. The pristine MoO_3_ sample, presented in Figure 3(a1–a3), displays a nanosheet-like morphology composed of irregularly stacked sheet structures. After the incorporation of PEDOT:PSS, noticeable changes in surface morphology are observed. For MoO_3_-PEDOT:PSS-1, shown in Figure 3(b1–b3), the MoO_3_ nanosheets are partially covered by a thin polymeric layer with fiber-like features. This indicates the successful deposition of PEDOT:PSS on the MoO_3_ surface. With further increase in PEDOT:PSS concentration, MoO_3_-PEDOT:PSS-2 in Figure 3(c1–c3) displays a more uniform and interconnected polymeric network distributed over the MoO_3_ nanosheet framework. The fiber-like PEDOT:PSS coating appears well integrated with the MoO_3_ surface, forming an open and porous hybrid architecture. This optimized morphology is beneficial for exposing more electrochemically active sites, shortening ion-diffusion pathways, and enhancing electron transport through the conductive PEDOT:PSS network. In contrast, MoO_3_-PEDOT:PSS-3, shown in Figure 3(d1–d3), exhibits a denser surface morphology due to excess PEDOT:PSS deposition. The higher PEDOT:PSS concentration appears to partially cover the MoO_3_ nanosheet surface and reduce the openness of the electrode structure. Such excessive polymer coverage may block some active sites and restrict electrolyte diffusion, leading to less efficient ion/electron transport compared with MoO_3_-PEDOT:PSS-2.

**Figure 4:**
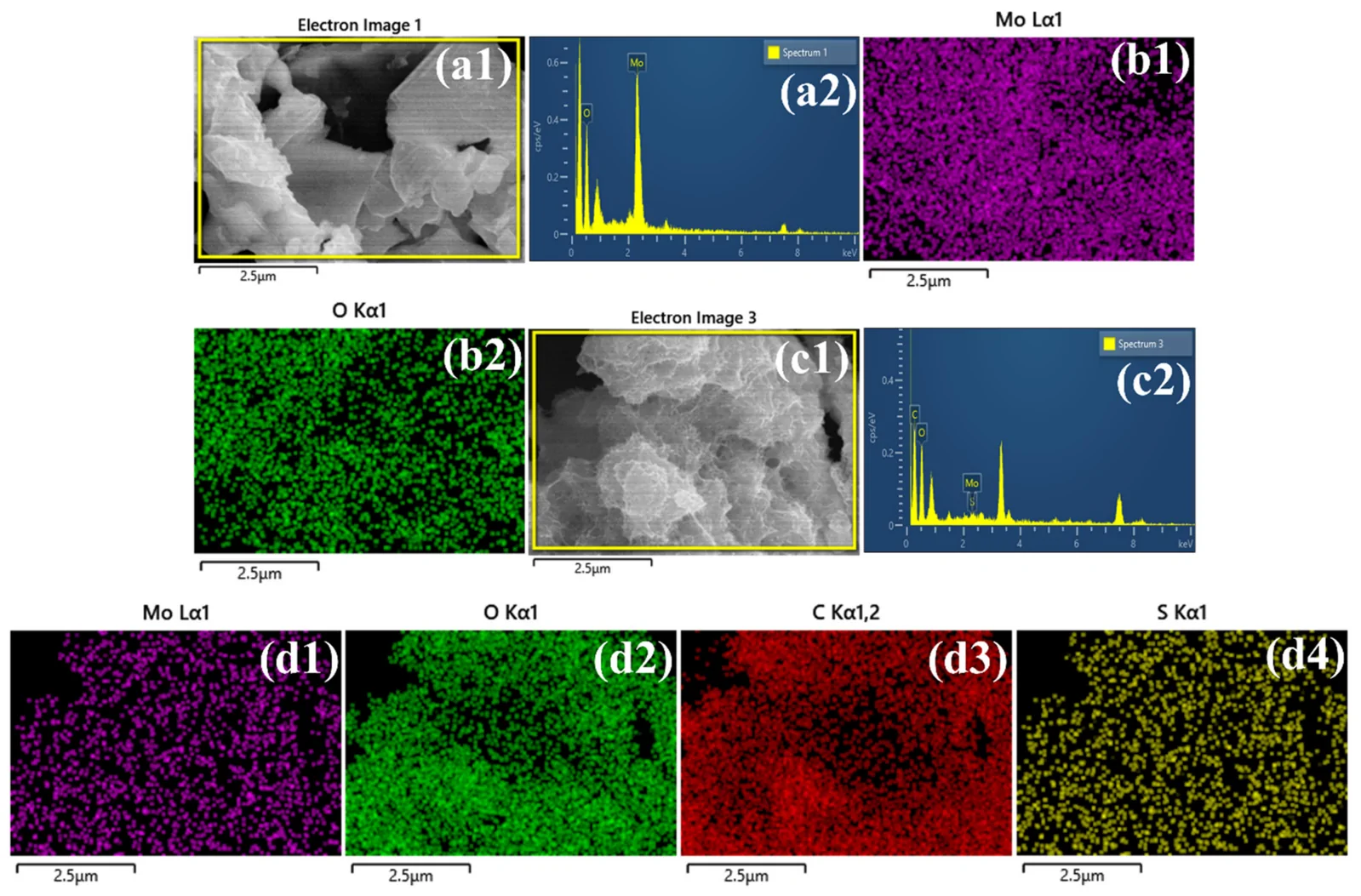
(**a1**,**a2**) EDX analysis, and (**b1**,**b2**) elemental mapping data of MoO_3_. (**c1**,**c2**) EDX analysis, and (**d1**–**d4**) elemental mapping data of MoO_3_-PEDOT:PSS-2.

Energy-dispersive X-ray spectroscopy (EDX) was conducted to determine the elemental composition and confirm the successful formation of MoO_3_ and the MoO_3_-PEDOT:PSS-2 composite, as revealed in Figure 4. The EDX spectrum of pristine MoO_3_ (Figure 4(a1,a2)) shows the existence of Mo and O elements, with elemental contents of 48.28 wt% and 51.72 wt%, respectively. For the optimized MoO_3_-PEDOT:PSS-2 composite, EDX spectrum are presented in Figure 4(c1,c2). In addition to Mo and O, distinct C and S signals are observed, confirming the incorporation of PEDOT:PSS onto the MoO_3_ surface. The elemental composition of MoO_3_-PEDOT:PSS-2 was found to be 15.56 wt% Mo, 38.59 wt% O, 40.96 wt% C, and 4.89 wt% S. Elemental mapping was further performed to evaluate the localized distribution of the constituent elements. As shown in Figure 4(b1,b2), Mo and O are uniformly distributed throughout the pristine MoO_3_ sample, indicating the homogeneous formation of the oxide structure. Similarly, the elemental mapping images of MoO_3_-PEDOT:PSS-2 (Figure 4(d1–d4)) disclose an even distribution of Mo, O, C, and S across the selected region. The homogeneous dispersion of C and S confirms the effective coverage of PEDOT:PSS over the MoO_3_ framework. Moreover, the uniform coexistence of Mo and PEDOT:PSS-related elements suggests strong interfacial contact between the crystalline MoO_3_ nanosheets and the conductive polymeric PEDOT:PSS network.

**Figure 5:**
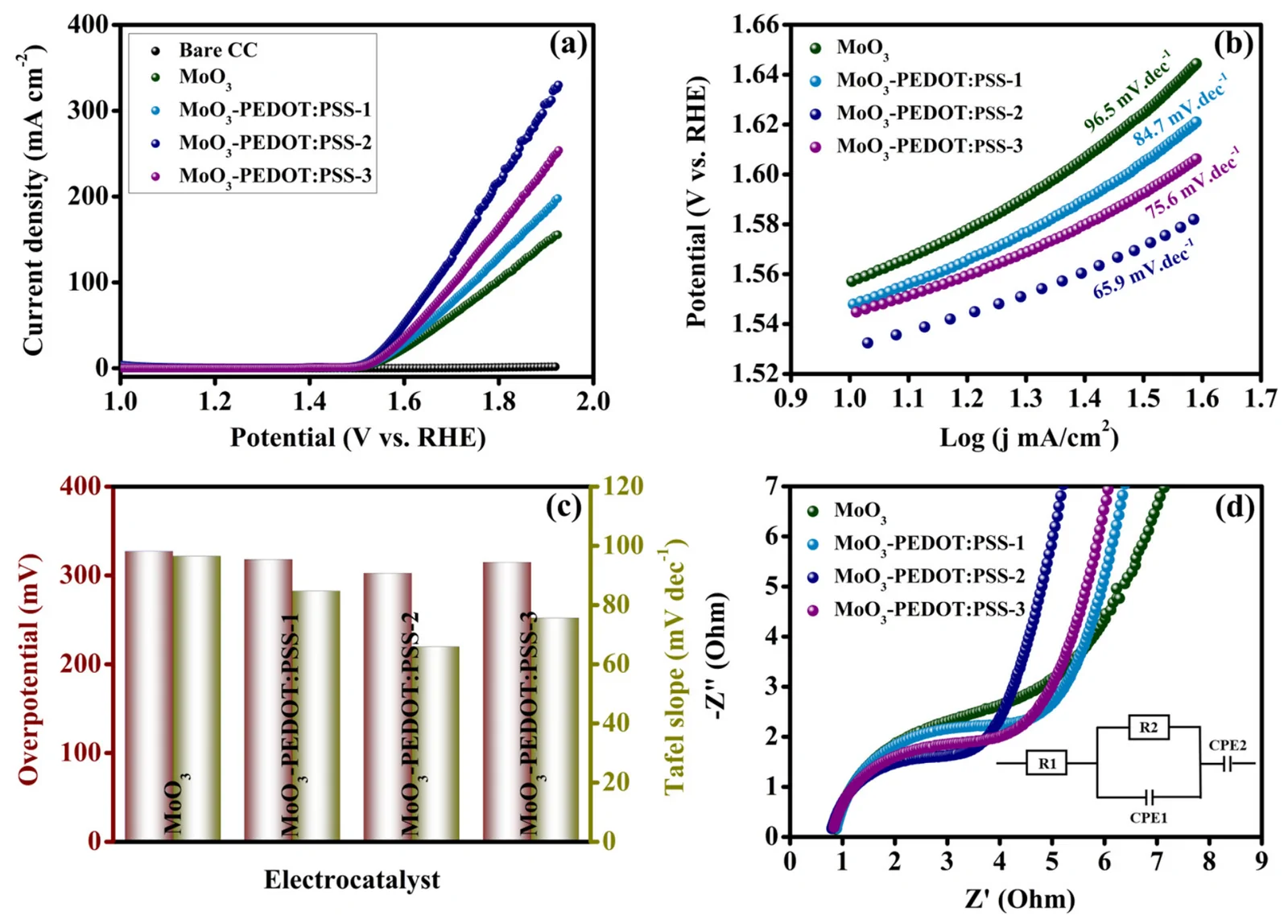
Electrochemical evaluation of the OER performance of the prepared electrocatalysts: (**a**) LSV curves recorded at a scan rate of 5 mV s^−1^, (**b**) Tafel plots, (**c**) comparison of overpotential at 10 mA cm^−2^ and Tafel slope, and (**d**) EIS spectra with the fitted equivalent circuit shown in the inset.

The oxygen evolution reaction (OER) activity of the prepared electrocatalysts was systematically evaluated in alkaline medium, where the overpotential vital to deliver a benchmark current density is a critical indicator of catalytic efficiency [52]. A lower overpotential reflects more favorable reaction energetics and enhanced catalytic activity. As shown in Figure 5a, all MoO_3_-PEDOT:PSS composite electrodes exhibited improved OER activity compared with pristine MoO_3_ and bare carbon cloth (CC), confirming the beneficial role of PEDOT:PSS incorporation. Among the investigated samples, MoO_3_-PEDOT:PSS-2 delivered the best catalytic performance, demanding an overpotential of only 302.4 mV to achieve a current density of 10 mA cm^−2^. This value is considerably lesser than those of pristine MoO_3_ (327.1 mV), MoO_3_-PEDOT:PSS-1 (317.9 mV), and MoO_3_-PEDOT:PSS-3 (314.8 mV) under identical testing conditions. The bare CC electrode exhibited negligible OER activity and failed to achieve a current density of 10 mA cm^−2^ within the investigated potential range, indicating its limited catalytic contribution. The superior activity of MoO_3_-PEDOT:PSS-2 can be attributed to the optimized PEDOT:PSS content by forming a conductive network with MoO_3_, which accelerates electron transfer and reduces charge-transfer resistance during OER. However, excessive PEDOT:PSS loading in MoO_3_-PEDOT:PSS-3 may partially block active sites or hinder ion diffusion, resulting in a slight decrease in catalytic performance compared with MoO_3_-PEDOT:PSS-2. To further understand the reaction kinetics, Tafel analysis was performed, as presented in Figure 5b. The calculated Tafel slopes for MoO_3_, MoO_3_-PEDOT:PSS-1, MoO_3_-PEDOT:PSS-2, and MoO_3_-PEDOT:PSS-3 were 96.5, 84.7, 65.9, and 75.6 mV dec^−1^, respectively. The knowingly reduced Tafel slope of MoO_3_-PEDOT:PSS-2 indicates faster OER kinetics and more effectual charge-transfer behavior at the electrode–electrolyte interface. This observation is reliable with the overpotential trend revealed in Figure 5c, further demonstrating the superior catalytic efficiency of the optimized composite electrode. The Tafel slope of 65.9 mV dec^−1^ suggests that the rate-determining step is likely associated with the transformation of adsorbed *O intermediates into *OOH species during the OER process [53]. To further evaluate the catalytic performance of the optimized MoO_3_-PEDOT:PSS-2 electrode, its OER activity was compared with previously reported Mo-based and conductive-polymer-modified electrocatalysts, as summarized in Table 1. Electrochemical impedance spectroscopy (EIS) was performed to evaluate the interfacial charge-transfer behavior of the synthesized electrocatalysts under OER conditions, as illustrated in Figure 5d. The attained Nyquist plots were fitted using an appropriate equivalent circuit to quantitatively determine the charge-transfer resistance (R_ct_). The fitted R_ct_ values for MoO_3_, MoO_3_-PEDOT:PSS-1, MoO_3_-PEDOT:PSS-2, and MoO_3_-PEDOT:PSS-3 were 15.4, 10.9, 4.6, and 7.3 Ω, respectively. Among all the electrodes, MoO_3_-PEDOT:PSS-2 unveiled the lowest R_ct_ value, signifying the most favorable charge-transfer behavior at the electrode–electrolyte interface. This reduced resistance confirms that the improved integration of PEDOT:PSS efficiently improves the electrical conductivity of MoO_3_ and accelerates electron transport during the OER process, thereby contributing to enhanced catalytic kinetics.

**Figure 6:**
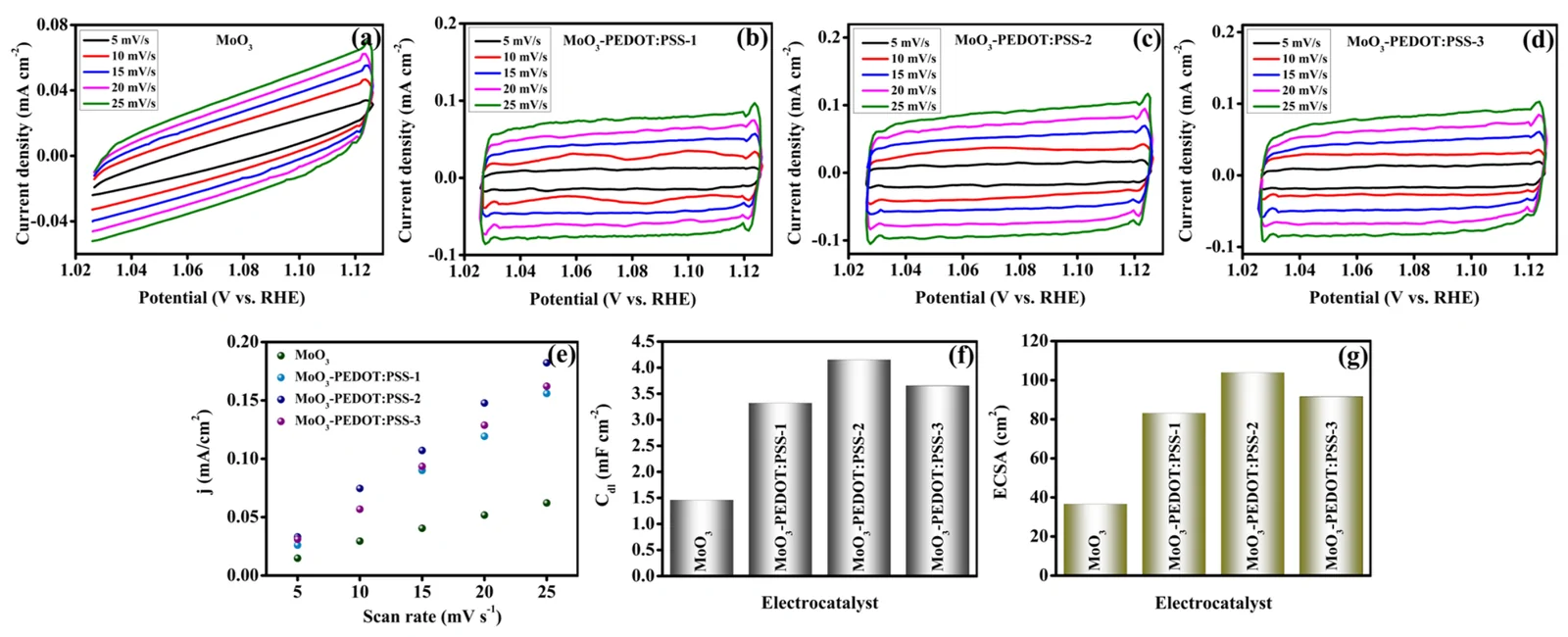
Cyclic voltammetry graph of (**a**) MoO_3_, (**b**) MoO_3_-PEDOT:PSS-1, (**c**) MoO_3_-PEDOT:PSS-2, and (**d**) MoO_3_-PEDOT:PSS-3 at diverse scan rate. (**e**) 2C_dl_ graph, (**f**) C_dl_ graph, and (**g**) ECSA outcomes of all the electrocatalyst.

Cyclic voltammetry measurements for MoO_3_, MoO_3_-PEDOT:PSS-1, MoO_3_-PEDOT:PSS-2, and MoO_3_-PEDOT:PSS-3 were conducted at various scan rates within the non-faradaic potential window, as presented in Figure 6a–d. In each case, the current density gradually increased with scan rate, indicating characteristic electrochemical double-layer capacitive behavior at the electrode/electrolyte boundary. To better correlate the OER activity with the availability of electrochemically accessible active sites, the electrochemically active surface area (ECSA) was determined using the double-layer capacitance (C_dl_). The corresponding 2C_dl_ and C_dl_ values, shown in Figure 6e and Figure 6f, respectively, were obtained from CV measurements recorded in the non-faradaic potential region. The estimated C_dl_ values for MoO_3_, MoO_3_-PEDOT:PSS-1, MoO_3_-PEDOT:PSS-2, and MoO_3_-PEDOT:PSS-3 were 2.93, 3.32, 4.15, and 3.65 mF cm^−2^, respectively. Consistently, the corresponding ECSA values were determined to be 36.62, 83.12, 103.87, and 91.62 cm^2^, respectively, as illustrated in Figure 6g. The highest C_dl_ and ECSA values obtained for MoO_3_-PEDOT:PSS-2 indicate that this electrode possesses the largest number of electrochemically accessible active sites. The enhanced ECSA of MoO_3_-PEDOT:PSS-2 can be attributed to the optimized PEDOT:PSS integration, which improves the electrode architecture by facilitating active-site exposure, strengthening interfacial contact, and promoting efficient ion/electron transport [54].

**Figure 7:**
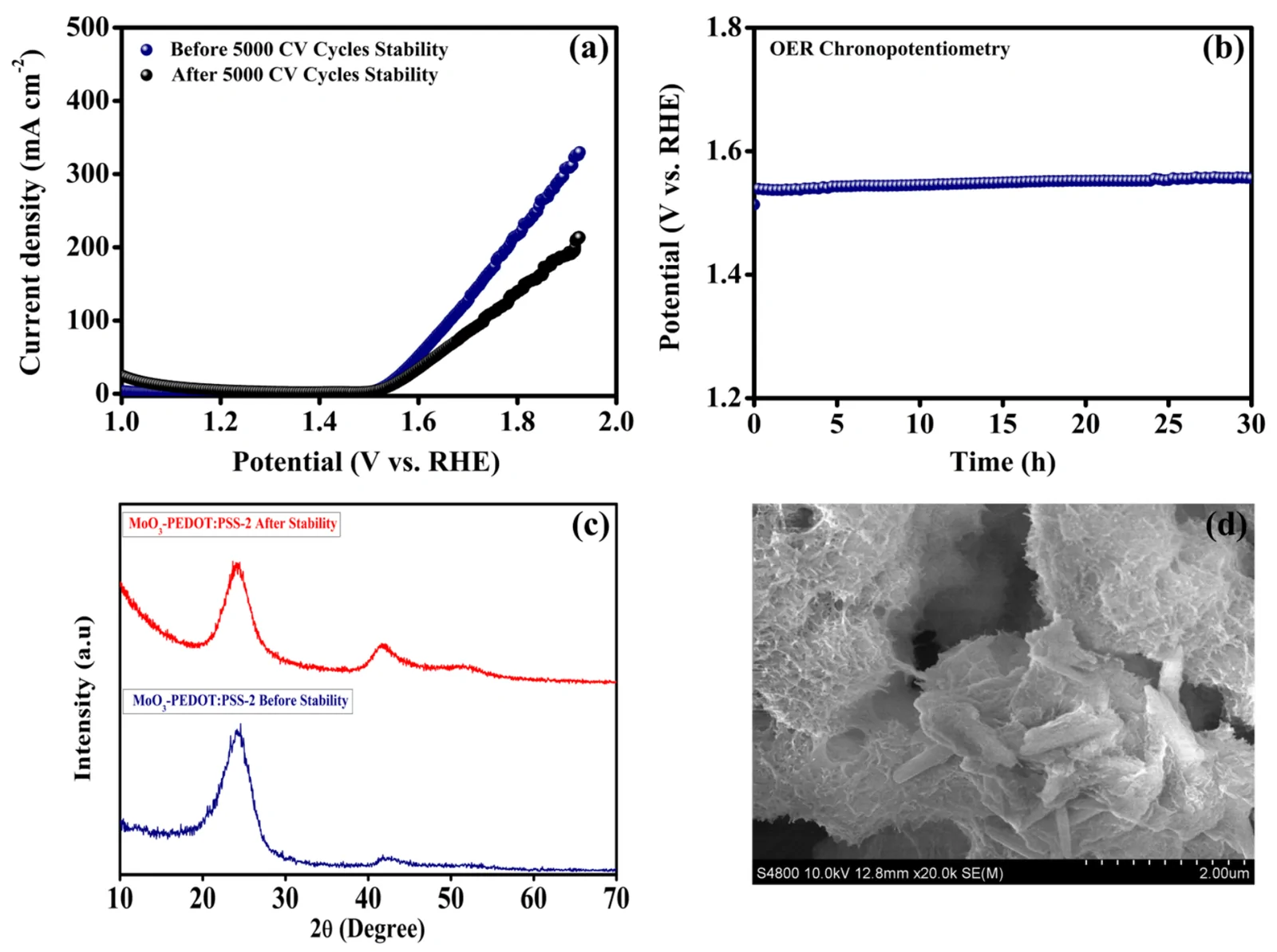
(**a**) LSV curves of MoO_3_-PEDOT:PSS-2 before and after 5000 CV cycles and (**b**) chronopotentiometry analysis. After stability (**c**) XRD pattern, and (**d**) FESEM image of MoO_3_-PEDOT:PSS-2.

The long-term operational stability of an electrocatalyst is a key criterion for evaluating its practical applicability in alkaline water oxidation. Therefore, the electrochemical durability of the augmented MoO_3_-PEDOT:PSS-2 electrocatalyst was investigated in 1.0 M KOH using accelerated cyclic voltammetry and chronopotentiometry. The accelerated durability test was executed by continuous CV cycling at a scan rate of 50 mV s^−1^ to study the stability of the catalyst under repeated electrochemical oxidation circumstances. As revealed in Figure 7a, the LSV curves of MoO_3_-PEDOT:PSS-2 were noted before and after 5000 CV cycles to evaluate the retention of OER activity. After prolonged cycling, the catalyst required an overpotential of 311.8 mV to sustain a current density of 10 mA cm^−2^, showing only a slight increase compared with the initial value. This minimal change in overpotential indicates that the MoO_3_-PEDOT:PSS-2 electrode maintains its catalytic activity and structural integrity during repeated OER operation. Although a noticeable decrease in current density is observed at higher applied potentials after 5000 CV cycles. This reduction may be associated with diminished accessibility or partial blockage of electrochemically active sites during prolonged electrochemical cycling. To further verify the steady-state stability, chronopotentiometry was directed at a constant current density of 10 mA cm^−2^ for 30 h, as presented in Figure 7b. The MoO_3_-PEDOT:PSS-2 electrocatalyst exhibited stable potential response over the continuous operation period, demonstrating good endurance under alkaline OER conditions. The slight performance fluctuation or minor loss observed during long-term operation may be associated with partial detachment or leaching of active species from the electrode surface, accumulation of generated oxygen bubbles at the electrode–electrolyte interface, and possible surface reconstruction or oxidation under high anodic potentials. Nevertheless, the overall stability results confirm that MoO_3_-PEDOT:PSS-2 possesses robust electrochemical durability, making it a promising electrocatalyst for alkaline oxygen evolution [55,56]. The structural and morphological characteristics of the optimized MoO_3_-PEDOT:PSS-2 electrode following the OER durability test were examined using XRD and FESEM analyses. As presented in Figure 7c, the post-stability XRD profile exhibits the principal diffraction features associated with MoO_3_, suggesting that the crystalline phase is largely retained under the investigated electrochemical conditions. A reduction in diffraction peak intensity is observed after durability testing; however, no obvious additional crystalline phases are identified within the detection limits of the XRD measurement. Furthermore, the post-stability FESEM image in Figure 7d reveals that the electrode largely maintains its characteristic nanosheet-like morphology and interconnected surface features. Although localized surface roughening and partial aggregation are noticeable, the overall morphology remains recognizable after electrochemical testing.

**Table 1:** Comparison of present electrocatalyst OER performance result in 1 M KOH with other reported electrocatalysts.

Materials	Overpotential (mV)	Current Density	Ref.
Co-doped MoO_x_	340	10 mA cm^−2^	[57]
Ag doped MoO_3_	330	10 mA cm^−2^	[58]
Cu(OH)_2_@MoO_3_	308	10 mA cm^−2^	[59]
MnO_x_-MoO_3_	390	10 mA cm^−2^	[60]
Co_2_P_2_O_7_@MoO_3_/NF	377	10 mA cm^−2^	[43]
Co-doped MoSe_2_@PANI	385	10 mA cm^−2^	[61]
Cu_2_MoS_4_-PANI/rGO	395	10 mA cm^−2^	[62]
NiFeLDH@PANi-CF	310	10 mA cm^−2^	[63]
CoFe_2_O_4_/PANI-MWCNTs	314	10 mA cm^−2^	[64]
Fe-Mn@PANI/NF	353	10 mA cm^−2^	[65]
NiFe_2_O_4_/Ni_3_Fe/PANI	320	10 mA cm^−2^	[66]
PPy/ZnWO_4_	507	10 mA cm^−2^	[67]
CoNiMn-LDH/PPy/RGO	369	10 mA cm^−2^	[68]
PPy/IL nanoparticles	583	10 mA cm^−2^	[69]
MoO_3_-PEDOT:PSS-2	302	10 mA cm^−2^	Present work

## 3. Experimental Section

### 3.1. Chemicals

Ammonium heptamolybdate tetrahydrate ((NH_4_)_6_Mo_7_O_24_·4H_2_O) was purchased from Sigma-Aldrich (St. Louis, MO, USA). Hydrochloric acid (HCl, extra pure) and ethanol (C_2_H_5_OH, 94.5%) were obtained from Duksan Chemicals (Ansan-si, Gyeonggi-do, Republic of Korea). Potassium hydroxide (KOH, ≥85%) was provided by Daejung Chemicals & Metals (Siheung-si, Gyeonggi-do, Republic of Korea). A commercially available PEDOT:PSS aqueous dispersion (Clevios™ PH 1000, Lot No. 2015P0052) was obtained from Heraeus (Leverkusen, Germany). The dispersion contained 1.3 wt.% PEDOT:PSS solids, with a PSS-to-PEDOT weight ratio of approximately 2.5:1. High-purity carbon cloth (CC), used as the conductive scaffold and current collector for electrode preparation, was purchased from NARA Cell-Tech Corporation (Seoul, Republic of Korea). All reagents used in this study were analytical-grade chemicals and were utilized directly without any additional purification.

### 3.2. Preparation of MoO_3_ and MoO_3_-PEDOT:PSS

The synthesis process of MoO_3_-PEDOT:PSS is illustrated in Figure 1. MoO_3_ was synthesized directly on carbon cloth by a hydrothermal method. In a representative synthesis, 1.4 g of ((NH_4_)_6_Mo_7_O_24_·4H_2_O) was added to 30 mL of deionized water and magnetically stirred for 20–30 min until a transparent and uniform solution was formed. Next, diluted HCl was introduced drop by drop to adjust the pH of the precursor solution to about 1–2. The acidic environment promotes the transformation of molybdate ions into molybdenum oxide or hydrated molybdenum oxide species during the hydrothermal reaction. The resulting solution was then transferred into a 50 mL Teflon-lined stainless-steel autoclave, and the pre-cleaned carbon cloth was vertically placed inside the Teflon liner, ensuring complete immersion in the precursor solution. The sealed autoclave was maintained at 180 °C for 12 h to complete the hydrothermal reaction. Following natural cooling to room temperature, the MoO_3_-coated carbon cloth was carefully removed and washed numerous times with deionized water and ethanol to disregard residual ions and loosely attached particles. The obtained sample was dried at 60 °C for 8–12 h and subsequently annealed in air at 350 °C for 2 h to obtain the final MoO_3_ electrode.

The MoO_3_-PEDOT:PSS composite electrodes were prepared by a simple immersion method. The as-synthesized MoO_3_-coated carbon cloth electrodes were individually immersed for 2 min in PEDOT:PSS modification solutions prepared by dispersing 0.05, 0.10, and 0.15 g of the as-received commercial PEDOT:PSS aqueous dispersion in 30 mL of ultrapure water, respectively. After immersion, the electrode was slowly withdrawn from the PEDOT:PSS dispersion, allowed to drain naturally to remove excess solution, and dried at 60 °C for 6 h to obtain PEDOT:PSS-modified MoO_3_. By varying the PEDOT:PSS concentration in the dispersion, different composite electrodes were obtained and denoted as MoO_3_-PEDOT:PSS-1, MoO_3_-PEDOT:PSS-2, and MoO_3_-PEDOT:PSS-3.

### 3.3. Material Characterization

The structural, chemical, and morphological assets of the synthesized materials were systematically characterized using advanced analytical techniques. X-ray diffraction (XRD) analysis was achieved on an X’Pert Pro diffractometer by means of Cu Kα radiation (λ = 1.5418 Å) to detect the crystalline phases, evaluate phase purity, and examine the structural characteristics of the prepared samples. X-ray photoelectron spectroscopy (XPS) was implemented using a Thermo Scientific K-Alpha spectrometer to examine the surface elemental composition, oxidation states, and chemical bonding environments. The surface morphology and microstructural structures were analyzed by field-emission scanning electron microscopy (FESEM, HITACHI S-4800). Moreover, energy-dispersive X-ray spectroscopy (EDX) coupled with FESEM was employed to regulate the elemental composition and spatial distribution of the integral elements within the synthesized materials.

### 3.4. Electrochemical Analysis

Electrochemical analyses were performed at room temperature on a Bio-Logic VSP-300 electrochemical workstation (Gières, France) using a standard three-electrode system in 1.0 M KOH electrolyte. The synthesized catalyst materials were grown directly on carbon cloth, which was used as the working electrode. An Hg/HgO electrode and a platinum plate were used as the reference and counter electrodes, respectively. Before catalyst preparation, the carbon cloth was thoroughly treated by ultrasonication in 1.0 M HCl, deionized water, and ethanol for 20 min individually to eliminate exterior impurities, subsequently dried overnight at 60 °C.

The OER performance of the fabricated electrodes was examined using LSV at a scan rate of 5 mV s^−1^. The recorded potentials were converted to the reversible hydrogen electrode (RHE) scale to enable reliable comparison of catalytic performance.

E_RHE_ = E_Hg/HgO_ + E°_Hg/HgO_ + 0.0591 × (pH) (1)

where E°_Hg/HgO_ signifies the standard potential of the Hg/HgO reference electrode under alkaline conditions (pH ≈ 14). The Tafel slopes were determined by linear regression of the overpotential versus logarithmic current density (η-log j) plots within the current density range of 10–40 mA cm^−2^. The electrochemical double-layer capacitance (C_dl_) was determined from cyclic voltammetry (CV) curves recorded in the non-Faradaic potential area of 0.1–0.2 V vs. Hg/HgO at different scan rates ranging from 5 to 25 mV s^−1^. The electrochemically active surface area (ECSA) was assessed from the obtained C_dl_ results using a specific capacitance (C_s_) of 0.040 mF cm^−2^ conferring to the following equation:

ECSA = Cdl/Cs(2)

Electrochemical impedance spectroscopy (EIS) was performed at an applied potential of 1.53 V over a frequency of 100 kHz to 0.1 Hz with an AC amplitude of 10 mV. The electrochemical resilience of the augmented electrocatalyst was assessed by accelerated cycling stability tests involving 5000 continuous CV cycles at a scan rate of 50 mV s^−1^. The LSV polarization curves verified before and after cycling were compared to evaluate activity retention. In addition, long-term operational stability was inspected by chronopotentiometry at a constant current density under continuous OER conditions.

## 4. Conclusions

In summary, a MoO_3_-PEDOT:PSS hybrid electrocatalyst was successfully developed on carbon cloth through hydrothermal growth of MoO_3_ followed by PEDOT:PSS surface modification using a simple immersion strategy. The structural and surface analyses confirmed the formation of crystalline MoO_3_ and the successful incorporation of PEDOT:PSS, where the conductive polymeric network effectively modified the MoO_3_ surface without disturbing its primary crystalline framework. Among the prepared electrodes, MoO_3_-PEDOT:PSS-2 exhibited the most favorable morphology, with an optimized nanosheet-polymeric-network architecture that promoted active-site exposure, electrolyte accessibility, and interfacial charge transport. The optimized MoO_3_-PEDOT:PSS-2 electrode delivered superior alkaline OER performance, requiring a low overpotential of 302.4 mV to achieve 10 mA cm^−2^, which was knowingly lower than those of pristine MoO_3_ (327.1 mV), MoO_3_-PEDOT:PSS-1 (317.9 mV), and MoO_3_-PEDOT:PSS-3 (314.8 mV). In addition, MoO_3_-PEDOT:PSS-2 exhibited the smallest Tafel slope of 65.9 mV dec^−1^, confirming faster OER kinetics. The enhanced catalytic activity was further supported by its reduced R_ct_ of 4.6 Ω, higher C_dl_ value of 4.15 mF cm^−2^, and enlarged ECSA of 103.87 cm^2^, demonstrating improved electrochemical accessibility and effective electron transport at the electrode–electrolyte interface. Furthermore, MoO_3_-PEDOT:PSS-2 shows reasonable activity after 5000 CV cycles, with an overpotential of 311.8 mV at 10 mA cm^−2^, and preserved stable operation during 30 h chronopotentiometry testing. The improved OER performance of MoO_3_-PEDOT:PSS-2 may be associated with the beneficial integration of MoO_3_ and conductive PEDOT:PSS, which facilitates interfacial charge transport, electrochemical accessibility, and favorable reaction kinetics.

## Data Availability

The data presented in this study are available on request from the corresponding author. (please specify the reason for restriction, e.g., the data are not publicly available due to privacy or ethical restrictions.)
